# Cytotoxic Effects of the Radiocontrast Agent Iotrolan and Anesthetic Agents Bupivacaine and Lidocaine in Three-Dimensional Cultures of Human Intervertebral Disc Nucleus Pulposus Cells: Identification of the Apoptotic Pathways

**DOI:** 10.1371/journal.pone.0092442

**Published:** 2014-03-18

**Authors:** Koji Iwasaki, Hideki Sudo, Katsuhisa Yamada, Manabu Ito, Norimasa Iwasaki

**Affiliations:** 1 Department of Orthopaedic Surgery, Hokkaido University Graduate School of Medicine, Sapporo, Japan; 2 Department of Advanced Medicine for Spine and Spinal Cord Disorders, Hokkaido University Graduate School of Medicine, Sapporo, Japan; University of Nebraska Medical Center, United States of America

## Abstract

**Background:**

Discography and discoblock are imaging procedures used to diagnose discogenic low back pain. Although needle puncture of the intervertebral disc (IVD) itself induces disc degeneration, the agents used in these procedures may also have harmful effects on IVD cells. The purpose of this study was to analyze whether radiocontrast agents and local anesthetic agents have detrimental effects on human nucleus pulposus (NP) cells.

**Methods:**

Healthy human NP cells were cultured for 7 days in three-dimensional (3D) cell–alginate bead composites, and were then exposed to clinically relevant doses of a radiocontrast agent (iotrolan) or local anesthetic (lidocaine or bupivacaine). Cell viability and apoptosis were measured by confocal microscopy and flow cytometry. On the basis of caspase expression profiles, the apoptotic pathways activated by the agents were identified by Western blot analysis.

**Results:**

The radiocontrast agent iotrolan did not affect NP cell viability or induce apoptosis. In contrast, both the anesthetic agents significantly decreased cell viability and increased the apoptotic cell number in a time- and dose-dependent manner. After 120 min, 2% lidocaine and 0.5% bupivacaine decreased percent live cells to 13% and 10%, respectively (p<0.05). The number of apoptotic cells was doubled by increasing lidocaine dosage from 1% to 2% (23% and 42%) and bupivacaine from 0.25% to 0.50% (25% and 48%) (p<0.05). Western blot analysis revealed that both anesthetic agents upregulated cleaved caspase-3 and caspase-8, whereas only bupivacaine upregulated cleaved caspase-9.

**Conclusions/Significance:**

The present study demonstrates that iotrolan does not affect the viability of healthy human NP cells. In contrast, the two anesthetic agents commonly used in discography or discoblock may cause extensive damage to IVDs by inducing apoptotic cell death.

## Introduction

Discogenic low back pain (LBP) is among the most common disabilities worldwide. This condition is generally caused by the progressive intervertebral disc (IVD) degeneration associated with aging or due to trauma [1]. The diagnostic modalities are usually radiography, computed tomography, and magnetic resonance imaging. However, the association between imaging findings and pain is not direct because a fraction of the patient population is asymptomatic [1].

Provocative discography is an imaging procedure routinely used to diagnose discogenic LBP on the basis of the level of pain induced by the injection of radiocontrast agents [2]. A needle is inserted into the nucleus pulposus (NP) under fluoroscopic guidance and a radiocontrast agent is injected to the center of the painful IVD. The mechanism of pain provocation is largely unknown, but it is hypothesized that pathological metabolites, extruded from the disc, irritate nerve fibers in the outer annulus fibrosus (AF) [3]. Alternatively, analgesic discography (discoblock) can also be used to diagnose discogenic LBP on the basis of the level of pain relief caused by the injection of a small amount of bupivacaine [4]. However, a recent prospective, matched cohort study that included patients with severe discogenic LBP revealed that discography was associated with more extensive IVD degeneration and herniation at a 10-year follow-up [5]. Discography using small-gauge needles and limited pressurization resulted in accelerated IVD degeneration. Animal studies confirmed that needle puncture and pressure cause IVD degeneration [6]–[8]. In addition, *in vitro* studies showed that bupivacaine [2], [9], [10] and the contrast agent iopamidol [6] have toxic effects on IVD cells *in vitro*.

Other agents frequently used in the treatment and/or diagnosis of LBP could potentially represent safer alternatives, such as the anesthetic lidocaine and the contrast agent iotrolan. However, the potential toxic effects of these agents on IVD cells have not been investigated. Therefore, the purpose of this study was to compare the cytotoxicity of bupivacaine, lidocaine, and iotrolan in three-dimensional (3D) cultures of healthy human NP cells.

## Materials and Methods

### NP cell culture conditions

Healthy intact human NP samples were obtained from five patients (mean age ± standard deviation, 14.8±5.2 years) [11], [12] who underwent anterior spinal fusion for adolescent idiopathic scoliosis. Before surgery, all IVDs were analyzed by magnetic resonance imaging and graded for degenerative changes using the Pfirrmann classification system [13]. All IVDs were grade 1, suggesting that all samples were healthy. We obtained informed consent from the next of kin, caretakers, or guardians on the behalf of the minors (< 20 years old) participants involved in this study and the consent was written and the documents were saved. The ethics committee of the Hokkaido University Graduate School of Medicine specifically approved this study.

The NP cells were isolated and cultured as previously described [11], [12] using an extended 4 hour enzymatic digestion step. Briefly, each gel-like NP was separated from AF using a dissecting microscope. The tissue specimens were placed in a complete culture medium containing Dulbecco's modified Eagle's medium (Sigma-Aldrich, St. Louis, MO, USA), supplemented with 10% fetal bovine serum (Nichirei Bioscience, Tokyo, Japan), 1% penicillin/streptomycin, and 1.25-µg/mL Fungizone (Life Technologies, Carlsbad, CA, USA). The preparations were washed twice by centrifugation (1,000 rpm; 3 min) and resuspended in Dulbecco's modified Eagle's supplemented with 0.25% collagenase. To isolate NP cells, the preparations were incubated in a shaking incubator (37°C; 4 h) and were then centrifuged twice (1,000 rpm; 3 min). The cells released from the matrix were placed in 10-cm tissue culture dishes and incubated in a humidified atmosphere of 5% CO_2_ at 37°C for 4 to 6 weeks. After two passages in the complete culture medium, the NP cells were dissociated from the monolayers and the isolated cells were resuspended in 1.2% low viscosity alginate solution (Sigma-Aldrich) at 4 × 10^6^ cells/mL. Beads were formed by dispensing the alginate/cell suspension drop-wise into a 102 mM CaCl_2_ solution via a 22-gauge needle attached to a syringe. After 10 min, the newly formed beads were washed once with sterile 0.9% saline solution followed by two washes with complete medium [14]. They were maintained for 7 days in the complete culture medium in an incubator (37°C/5% CO_2_) before the experiments.

### Experimental protocol

After 7 days of culture, the NP cell–alginate 3D composites were placed in 24-well plates (5 beads per well) and exposed (30–120 min) to clinically relevant doses of the radiocontrast agent iotrolan (0.5 mL; 240 mg I/mL; Bayer Health Care AG, Leverkusen, Germany) or one of the two local anesthetic agents, i.e., 1%–2% lidocaine (AstraZeneca, Luton, UK) or 0.25%–0.50% bupivacaine (AstraZeneca). A 0.9% saline solution (Otsuka Pharmaceutical Co., Tokyo, Japan) was used as a control and NP cell-alginate 3D composites that were not exposed to any reagent were used as untreated controls. After the exposure, the NP cell–alginate 3D composites were washed twice with Hank's balanced salt solution and incubated in the complete culture medium for 24 h.

### Cell viability analysis

Live and dead cells were detected with calcein AM and propidium iodide (PI) (Dojindo, Kumamoto, Japan), respectively. Twenty-four hours after exposure to each agent, the NP cell–alginate 3D composites were stained with 5-µM calcein AM and 1.5-µM PI. Calcein AM emits a green fluorescence in live cells, whereas PI emits red fluorescence in dead cells. The stained composites were washed with Hank's balanced salt solution and examined by confocal laser scanning microscopy (Olympus Fluoview FV300, Tokyo, Japan). The number of live and dead cells among the beads was manually counted. Four fields, each containing at least 15 cells, were counted in each area [15].

### Analysis of apoptosis

Twenty-four hours after exposure to each agent, the NP cell–alginate 3D composites were dissolved in 55-mM sodium citrate at 4°C until the beads released the cells, which were collected by centrifugation. The NP cells were washed twice in phosphate-buffered saline, counted (3.6 × 10^5^), and labeled with the Annexin V-fluorescein isothiocyanate (FITC) Apoptosis Detection Kit II (BD Biosciences, San Jose, CA, USA) according to the manufacturer's instructions. Both early apoptotic cells (FITC+/PI−) and late apoptotic cells (FITC+/PI +) were monitored with a flow cytometer (FACS Cant; BD biosciences, CA, USA). The present study included both early and late apoptotic cells, and the results of both groups of cells were combined in the analysis [11], [16], [17].

### Western blot analysis

Western blot analysis was used to identify the apoptotic pathways activated by the agents in the NP cells. Twenty-four hours after exposure to each agent, the NP cells were retrieved from the NP cell–alginate 3D composites as described above, and total protein was obtained by lysing the cells in tissue protein extraction reagent (Thermo Scientific, Waltham, MA, USA). Cell lysates (20 μg protein/lane) were loaded and separated on a 4%–12% gradient polyacrylamide gel, then transferred to a polyvinylidene difluoride membrane by standard electroblotting. The blots were blocked with 5% nonfat milk containing 0.3% Tween 20 (Bio-Rad Laboratories, Hercules, CA, USA) for 1 h and incubated overnight with primary antibodies against caspase-3 (catalog number, 9665; Cell Signaling Technology, Danvers, MA, USA), caspase-8 (catalog number, ab32125; Abcam, Cambridge, MA, USA), caspase-9 (catalog number, ab32539; Abcam), cleaved caspase-3 (catalog number, 9665; Cell Signaling Technology), cleaved caspase-8 (catalog number, sc-7890; Santa Cruz Biotechnology, Santa Cruz, CA, USA), and cleaved caspase-9 (catalog number, ab32539; Abcam). The membranes were washed three times with Tris-bufferedsaline-Tween20 solution and further incubated with horseradish peroxidase-conjugated anti-rabbit IgG (catalog number, 7074; Cell Signaling Technology) secondary antibody for 1 h. The bands were detected with the ECL plus reagent (Invitrogen Carlsbad, CA, USA) using the ChemiDoc™ MP Imaging System (Bio-Rad Laboratories, Hercules, CA, USA). β-actin was used as an internal control to confirm equal protein loading.

### Statistical analysis

All data were expressed as mean ± standard error (SE). Five independent experiments were analyzed for each group and each time point. Statistical analyses were performed using one-way analysis of variance followed by a Bonferroni's post-hoc test for multiple group comparisons or unpaired Student's t-tests for two-group comparisons. A p value of <0.05 was considered statistically significant.

## Results

### Time- and dose-dependent effects of radiocontrast agents and local anesthetic agents on the viability of NP cells

**Figure 1:**
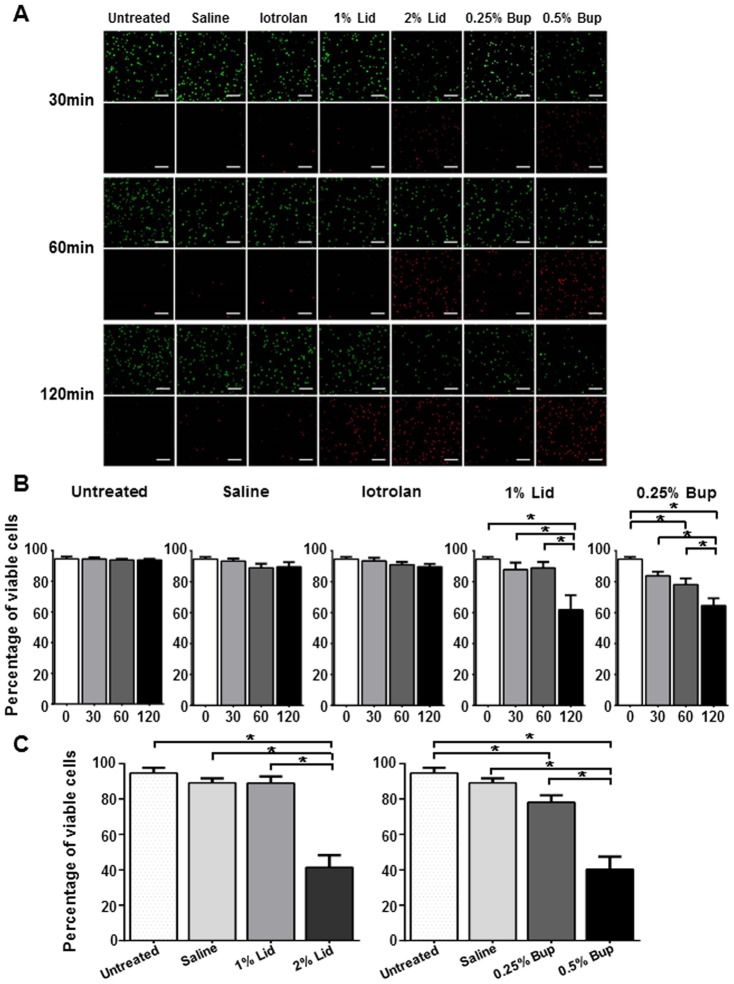
Impact of radiocontrast agents and local anesthetic agents on the viability of human nucleus pulposus (NP) cells. NP cell–alginate 3D composites were exposed to saline, radiocontrast agent (iotrolan) or local anesthetic agents (lidocaine and bupivacaine) for 30, 60, or 120 min. After an additional 24 h, they were stained with calcein AM (live cells; green) and propidium iodide (PI; dead cells; red). (**A**) Confocal laser scanning micrographs of the NP cell–alginate 3D composites after the exposures. Scale bar: 100 μm. (**B**) Time-dependent effects on cell viability. (**C**) Dose-dependent effects on viability after 60 min. All data are from five independent experiments (mean ± SE; *, p<0.05). Bup, bupivacaine; Lid, lidocaine.

We studied the effects of radiocontrast agents and local anesthetic agents routinely used during discoblock on the viability of healthy intact human NP cells grown in 3D cultures. The NP cell–alginate 3D composites were exposed to saline (control), iotrolan (radiocontrast agent), 1%–2% lidocaine, or 0.25%–0.50% bupivacaine (local anesthetic agents). Cell viability was measured after 30–120 min by calcein AM (live cells; green) and PI (dead cells; red) staining visualized by confocal microscopy (**Figure 1A**). Quantitative analysis showed that all NP cultures initially presented consistent viabilities in the 85%–90% range (**Figure 1B**). Exposure to saline or iotrolan did not affect cell viability. In contrast, both anesthetic agents exhibited a time-dependent cytotoxicity, with significant effects detected within 120 min (p<0.05). After 120 min, 1% lidocaine and 0.25% bupivacaine had decreased cell viability to 60% and 63%, respectively. Furthermore, both anesthetic agents induced a dose-dependent decrease in NP cell viability (**Figure 1C**). After 60 min, 1% lidocaine did not have any significant effect, whereas 2% lidocaine decreased cell viability to 40% (p<0.05). Likewise, the lowest concentration of bupivacaine did not affect viability, whereas 0.50% bupivacaine decreased cell viability to 39% (p<0.05).

**Figure 2:**
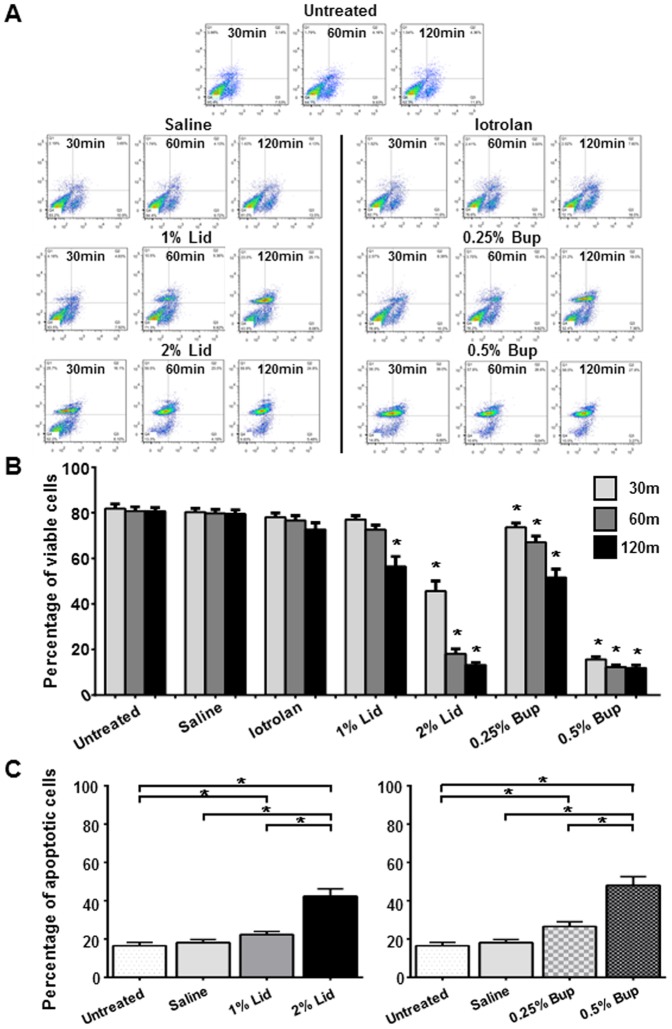
Flow cytometric analysis of human nucleus pulposus (NP) cells. NP cell–alginate 3D composites were exposed to saline, radiocontrast agent (iotrolan), or local anesthetic agents (lidocaine and bupivacaine) for 30, 60, or 120 min, cultured 24 h, then the NP cells were isolated for analysis. (**A**) Flow cytometry scatter plots. (**B**) Time- and dose-dependent effects on cell viability (*, p<0.05 vs. untreated control and saline at the same exposure time). (**C**) Dose-dependent effects on cell apoptosis after 60 min (*, p<0.05). Data are from five independent experiments (mean ± SE). Bup, bupivacaine; Lid, lidocaine.

These agents exhibited similar effects on the viability of the NP cells when they were extracted from the 3D cultures after the exposures and were labeled with Annexin V-FITC (**Figure 2A** **).** The percent viability counts obtained under control saline conditions were consistently in the 78%–80% range (**Figure 2B**). First, flow cytometry analysis showed that saline and iotrolan did not significantly affect NP cell viability during the 30–120 min exposures. Second, both anesthetic agents displayed time-dependent cytotoxic effects on the NP cells. After 120 min of exposure, 2% lidocaine and 0.5% bupivacaine decreased the percentage of live cells to 13% and 10%, respectively (p<0.05). Third, the anesthetic agents also exhibited dose-dependent cytotoxicity after 60-min exposures (**Figure 2C**). Increasing lidocaine dosage from 1% to 2% almost doubled the number of apoptotic cells from 23% to 42% (p<0.05). Similarly, raising bupivacaine concentration from 0.25% to 0.5% doubled the number of apoptotic cells, with 25% and 48%, respectively (p<0.05).

### Western blot analysis of the apoptotic pathways

**Figure 3:**
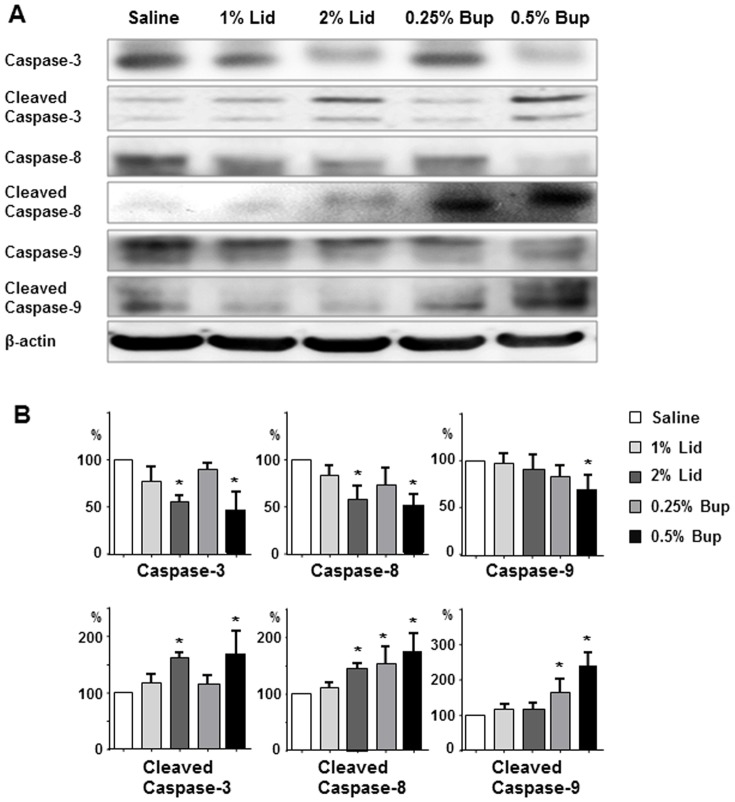
Western blot analysis of the apoptosis signaling pathways stimulated by lidocaine and bupivacaine. NP cell–alginate 3D composites were exposed 60 min to saline or a local anesthetic agent (lidocaine or bupivacaine), cultured 24 h, then the NP cell protein lysates were processed by sodium dodecyl sulfate polyacrylamide gel electrophoresis and immunoblotting with primary antibodies against caspase-3, caspase-8, caspase-9, cleaved caspase-3, cleaved caspase-8, and cleaved caspase-9. β-actin was used as an internal control. (**A**) Representative Western blot. (**B**) Densitometry analysis performed via normalization to β-actin. Data are from five independent experiments (mean ± SE; *, p<0.05 vs. saline).

The apoptotic signaling pathways implicated in the cytotoxicity of the local anesthetic agents were identified by Western blot analysis of key proteins (caspase-3, caspase-8, caspase-9, cleaved caspase-3, caspase-8, and caspase-9) after a 60 min exposure to varying concentrations of lidocaine and bupivacaine. Both anesthetic agents caused a dose-dependent upregulation of cleaved caspase-3 (**Figure 3A and 3B**). Furthermore, lidocaine upregulated cleaved caspase-8, whereas bupivacaine upregulated cleaved caspase-8 and caspase-9 (**Figure 3A and 3B**). These data suggest that each anesthetic agent triggers distinct patterns of apoptotic responses.

## Discussion

Discography and discoblock are representative procedures used to diagnose discogenic LBP. However, the potential toxic effects of the agents used in these procedures on human IVD cells have not been fully understood.

Over the years, new generations of radiocontrast agents were developed because their chemical properties, such as osmolarity, were found to considerably affect the severity of the side-effects [18]. Although the first generation had a high osmolarity relative to blood (>290 mOsm/kg H_2_O), the new generation contains primarily near- or low-osmolarity contrast agents, including nonionic dimeric iotrolan and nonionic monomeric iopamidol. In the present study, iotrolan did not exhibit any cytotoxic effect on human healthy NP cells. In contrast, Gruber et al. [6] reported that human AF cells exposed to iopamidol exhibited significant reduction in proliferation and increase in apoptosis compared with untreated cultures. This discrepancy is consistent with a clinical study showing that iotrolan was a safer and more efficient radiocontrast agent than iopamidol for the spinal cord [19]. The relatively low cytotoxicity of iotrolan was also shown in a study comparing the effects of inhaled iotrolan and iopamidol on rat lungs [20], in which iotrolan induced no significant organ or tissue toxicity, whereas iopamidol raised water and hemoglobin content and decreased blood PO_2_. Finally, higher tolerance to injection with iotrolan than iopamidol was reported in animal studies [21] and clinical trials [22]. Therefore, iotrolan should be the radiocontrast agent of choice for safe and effective diagnosis and treatment of IVD disorders.

Bupivacaine and lidocaine are common anesthetic agents used worldwide for the control of LBP in interventional techniques. However, there is currently no consensus on safe and effective dosages among physicians. Therefore, further comparisons on their cytotoxic effects on IVD cells remain to be made. The present study shows that clinically relevant doses of bupivacaine and lidocaine induced time- and dose-dependent decreases in viability and increases in the number of apoptotic cells among healthy human NP cells grown in 3D cultures. These data are consistent with a recent animal study showing that both anesthetic agents induced apoptosis in AF and NP cell monolayers from rabbit IVDs [23]. Our data are also consistent with *in vitro* studies conducted on IVD cells extracted from patients with IVD disorder. First, Lee et al. [9] reported the time- and dose-dependent cytotoxicity of bupivacaine in IVD cells from surgical specimens grown in monolayers (AF) or alginate beads (NP). Second, the cytotoxicity of this drug was confirmed in IVD-cell monolayers from human IVD surgical specimens [2]. The present study also shows that the cytotoxic effects of bupivacaine were comparable with those of lidocaine, which has received less attention in the literature regarding IVD disorders. The only related study suggested that lidocaine was more toxic than bupivacaine in rabbit AF and NP monolayers [23]. Consequently, we provide the first comparison of bupivacaine and lidocaine cytotoxicity in human IVD cells. The viability, apoptosis, and caspase analyses do not support a difference in the toxicity of these two anesthetic agents, which may reflect interspecies differences. Therefore, these observations indicate that local anesthetic agents used to control pain may negatively impact on human NP cells. In clinical settings, lidocaine's duration of action is approximately 50% that of bupivacaine, with lower reported systemic toxicity [24]. Therefore, lidocaine may be favored over bupivacaine for short procedures.

In this study, the flow cytometry results showed that approximately 20% of the cells underwent apoptosis following saline treatment for 60 min. Western blot analysis also showed some cleavage of caspase-3 in the saline-treated control cells. Apoptosis may have occurred during the sample preparation techniques used for each analytical method. However, the present study clearly showed that 2% lidocaine and 0.5% bupivacaine significantly induced NP cell apoptosis compared with saline-treated controls. Apoptosis plays a central role in the homeostasis of all tissues during normal development and tissue turnover. Two major signaling pathways control apoptosis through the activation of caspases in mammalian cells. The extrinsic pathway is activated via cell surface death receptors belonging to the tumor necrosis factor receptor superfamily, leading to caspase-8 activation. In contrast, the intrinsic pathway is initiated in the mitochondria by an intracellular injury, leading to caspase-9 activation. Mitochondria stressed by DNA damage or hypoxia release activated caspase-9 and its downstream effector caspase-3. Furthermore, caspase-8 cleavage mediates crosstalk between the death receptor and mitochondrial pathways [17], [25], [26]. Thus, analysis of caspase cleavage presented a simple and effective method to investigate the activation of these two major signaling pathways. In the present study, the anesthetic agents generated distinct protein expression patterns on Western blot analysis. Lidocaine upregulated cleaved caspase-3 and caspase-8, but bupivacaine upregulated cleaved caspase-3, caspase-8, and caspase-9. These results suggest that lidocaine activated the extrinsic pathway directly and possibly the intrinsic pathway by crosstalk via cleaved caspase-8 signaling. In contrast, bupivacaine may be able to directly activate both pathways. Degenerating NP cells have been shown to undergo apoptosis through mitochondrial involvement [17], [27]. Therefore, bupivacaine may exacerbate intrinsic apoptosis in degenerating NP cells, and initiate the extrinsic pathways as well. Because apoptosis plays an important role in the reduction of disc cell number during aging and IVD degeneration [17], [27], [28], the anesthetic agents used for discoblock should be carefully selected for low toxicity toward NP cells to minimize tissue degeneration.

There were some limitations to this study. First, there was a discrepancy between the numbers of viable cells recorded following 60 min of treatment of the two anesthetics at the higher dosages. Using calcein AM and PI staining resulted in a % viability of approximately 40% for both anesthetics, whereas the annexin V and PI staining used with flow cytometry resulted in a viability of approximately 20% for the two anesthetics. This discrepancy may have been due to differences in the sample preparation techniques used for each analytical method. Second, the main limitation to this study was that the *in vitro* data may not necessarily reflect the response of patients with IVD diseases in clinical settings. Because the exposure times used in this study were derived from published reports that investigated the *in vitro* toxicity of contrast media or local anesthetic on intervertebral disc cells [2], [9], we cannot relate these exposure times to clinical applications. Furthermore, we cannot comment on the average time that these chemicals would normally come in contact with IVD cells in clinical situations. However, it has been shown that cell viability after exposure to bupivacaine is greater in intact bovine articular cartilage compared with that of chondrocytes suspended in alginate, suggesting that the natural native matrix structure may provide some protection from local anesthetic exposure [29]. The NP cells from healthy human IVD cells may be less susceptible to anesthetic agents than those from degenerated IVDs. Most studies conducted on tissue biopsies from degenerating human IVDs examined cells from the outer AF [2], [6], [9], [10] because it is extremely difficult to distinguish NP from AF tissue. In addition, we have recently initiated *in vivo* studies in animal models of IVD degeneration to determine the long-term effects of bupivacaine on IVD degeneration.

In summary, the present study demonstrates that iotrolan should be selected over other common radiocontrast agents (i.e., iopamidol) to minimize toxicity-related IVD damage during discography. Moreover, the dosage of local anesthetic agents should be decreased to a minimum because increasing bupivacaine concentration from 0.25% to 0.50% decreased cell viability by 50%, and induced severe apoptotic cell death. Finally, the use of lidocaine as an alternative anesthetic agent does not appear promising because clinically relevant doses caused IVD cell damage comparable with bupivacaine. These results provide a basis for further *in vivo* studies to determine the long-term effects of local anesthetic agents on IVD degeneration.
